# Trehalose-Induced Regulations in Nutrient Status and Secondary Metabolites of Drought-Stressed Sunflower (*Helianthus annuus* L.) Plants

**DOI:** 10.3390/plants11202780

**Published:** 2022-10-20

**Authors:** Firdos Kosar, Khalid S. Alshallash, Nudrat Aisha Akram, Muhammad Sadiq, Muhammad Ashraf, Dalal Hussien M. Alkhalifah, Arafat Abdel Hamed Abdel Latef, Amr Elkelish

**Affiliations:** 1 Department of Botany, Government College University Faisalabad, Faisalabad 38040, Pakistan; 2 College of Science and Humanities–Huraymila, Imam Mohammed Bin Saud Islamic University (IMSIU), Riyadh 11432, Saudi Arabia; 3 Institute of Molecular Biology & Biotechnology, University of Lahore, Lahore 54590, Pakistan; 4 Department of Biology, College of Science, Princess Nourah Bint Abdulrahman University, Riyadh 11671, Saudi Arabia; 5 Department of Botany and Microbiology, Faculty of Science, South Valley University, Qena 83523, Egypt; 6 Botany Department, Faculty of Science, Suez Canal University Ismailia, Ismailia 41522, Egypt

**Keywords:** drought stress, trehalose, sunflower, exogenous application, enzymatic antioxidants

## Abstract

Trehalose regulates key physio-biochemical parameters, antioxidants, and the yield of plants exposed to a dry environment. A study was conducted to assess the regulatory roles of exogenously applied trehalose in drought-stressed sunflower plants. Two cultivars of sunflowers (Hysun 33 and FH 598) were subjected to drought stress (60% field capacity) and varying (0, 10, 20, and 30 mM) concentrations of trehalose. The data indicated that water stress significantly reduced the shoot length, root length, total soluble proteins, shoot Ca^2+^, root P, relative water content (RWC), and achene yield per plant. The foliar spray of trehalose was effective at improving plant growth, RWC, total soluble proteins, total soluble sugars, the activities of enzymatic antioxidants, Ca^2+^ (shoot and root), root K^+^, and the yield attributes. Exogenously supplemented trehalose considerably suppressed relative membrane permeability (RMP), but did not alter ascorbic acid, malondialdehyde, the total phenolics, shoot K^+^, or P (shoot and root) in both sunflower cultivars. The cv. Hysun 33 had better ascorbic acid, total soluble sugars, non-reducing sugars, shoot P, and root P than the other cultivar, whereas cv. FH 598 was relatively better at regulating RMP, malondialdehyde, peroxidase, and root Ca^2+^ concentration. Overall, exogenously supplemented trehalose, particularly at 10 mM, was effective at improving the physiochemical parameters and yield of sunflower plants under stress conditions. Therefore, a better performance of sunflower cv. Hysun 33 under drought stress can be suggested as a trehalose-induced enhancement of yield and oxidative defense potential.

## 1. Introduction

Drought stress is known to induce osmotic stress in plants, which in turn causes considerable alterations in various plant physio-biochemical and molecular mechanisms [1,2]. Of several processes, the generation of reactive oxygen species (ROS) is a well-known process that occurs frequently in plants exposed to drought, ultimately causing oxidative stress in different organelles of a plant cell. The stress-induced oxidative stress leads to an altered lipid composition of biological membranes and altered cell redox regulatory functioning [3]. Enhanced activities/levels of antioxidants, both enzymatic and non-enzymatic, along with reduced levels of ROS such as hydrogen peroxide (H_2_O_2_) and malondialdehyde (MDA) are adaptive regulatory components of plant metabolism under arid conditions [4,5]. Moreover, plants accumulate organic osmolytes, e.g., sugars, phenolic compounds, glycine betaine, proline, ascorbic acid, etc. [6]. An excessive amount of these osmolytes safeguards the plant’s metabolic machinery from dehydration injuries [7]. Under water shortage events, serious losses to plant growth processes occur, and the cell division, cell proliferation, and cell maturation phases are markedly disturbed. Photosynthetic pigments deteriorate under extreme water stress conditions, consequently causing limited crop yield [6].

It has been noticed that little success has been achieved with transgenic plants for the overproduction of compatible osmolytes against stressful environments, but better results in terms of reasonable crop yields have been achieved by the use of different organic solutes as an exogenous application [8]. A variety of organic compounds are currently in practice to ameliorate the negative effects of stress, and trehalose is a vital organic substance that has shown significant growth enhancement in plants under stress [9]. Its high concentration in drought-tolerant plants shows a beneficial impact on biological membranes and other vital metabolites [10]. When applied to the foliage of plants, it is readily absorbed and translocated to whole plant tissues [11]. An exogenous application of trehalose improved the achene yield and oil composition of sunflower plants subjected to drought stress conditions [9]. Likewise, the exogenous application of a plant growth regulator, salicylic acid, improved the salinity and drought stress tolerance of *Vicia faba* plants [2]. The foliage spray of trehalose causes bio-regulatory effects on growth and photosynthetic pigments, and it also acts as a signaling molecule [12].

The sunflower is the world’s fourth most cultivated seed oil crop and its demand is expanding rapidly due to its unique oil fatty acid profile [13]. Sunflower oil contains valuable nutrients, including antioxidants, which safeguard the cells from oxidation. As sunflower oil has a very low quantity of cholesterol, it is safe for use, especially for older people [14]. This crop is sensitive to saline and drought conditions, as extreme stress events reduce its yield by up to 60% [15]. Therefore, the current study aimed to assess the influence of foliar-applied trehalose on the plant growth, osmoregulation, oxidative defense potential, ion homeostasis, and yield production of sunflowers subjected to drought stress conditions.

## 2. Results

**Figure 1:**
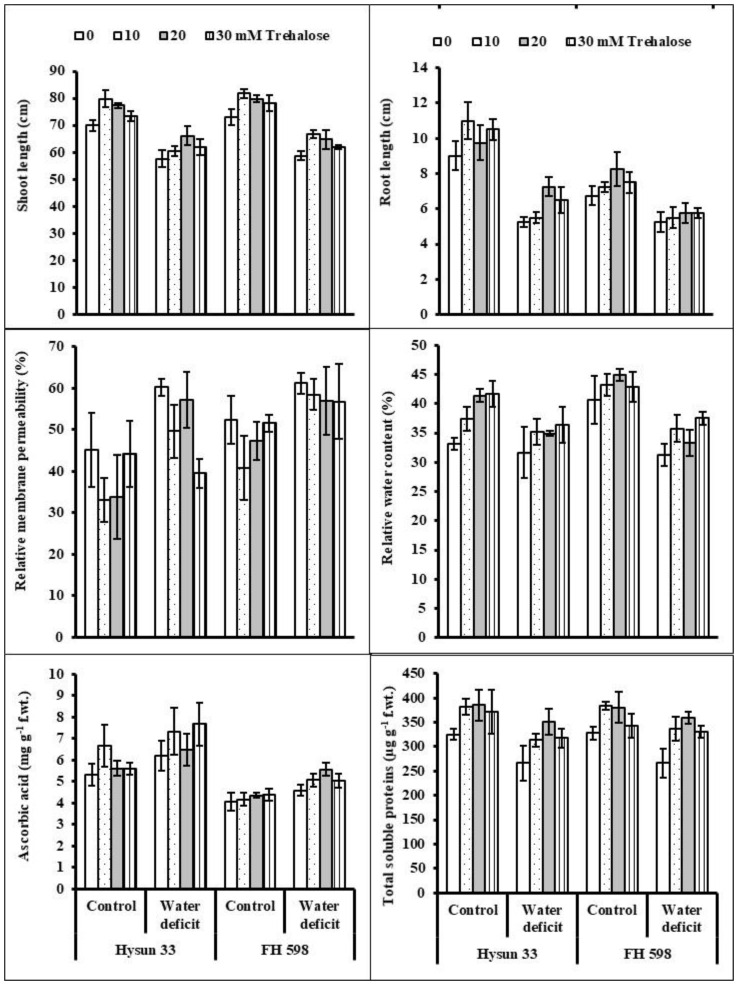
Shoot length, root length, relative membrane permeability, relative water content, ascorbic acid, and total soluble proteins of two drought-stressed and non-stressed cultivars of sunflower (*Helianthus annuus* L.) plants subjected to varying levels of foliar-applied trehalose (mean ± S.E.).

Drought stress reduced the length (shoot and root) of both sunflower cultivars. A positive effect of trehalose applied as a foliage spray was observed on the shoot length and root length of sunflower plants under both water regimes. Of the different concentrations of trehalose, 10 and 20 mM were better than the other levels at enhancing these growth attributes. Of both cultivars, cv. FH 598 was inferior to cv. Hysun 33 in shoot and root length under water stress (Figure 1).

A considerable increase was determined in the relative membrane permeability (RMP) of both sunflower cultivars due to water stress. An application of trehalose considerably suppressed the RMP of both cultivars and the most effective level of trehalose was found to be 10 mM. The difference between both sunflower cultivars was prominent in terms of RMP, and cv. FH 598 was higher in RMP at both water regimes (Figure 1).

The relative water content (RWC) was suppressed significantly in both sunflower cultivars under water stress. Foliar-applied trehalose (20 mM) was very effective at improving the RWC of both sunflower cultivars (Table 1; Figure 1). Of both sunflower cultivars, cv. FH 598 had a relatively better RWC under non-stress conditions, while both cultivars responded similarly under drought stress conditions.

Water stress induced a significant increase in the ascorbic acid (AsA) content of both sunflower cultivars. However, no significant effect of trehalose was noticed on the AsA content of either sunflower cultivar. The cv. Hysun 33 had a considerably better AsA content than cv. FH 598 under arid conditions.

Drought stress significantly suppressed the total soluble protein (TSP) content of both sunflower cultivars. The application of trehalose, particularly at the rates of 10 and 20 mM, enhanced the TSPs of stressed and non-stressed sunflower plants (Table 1; Figure 1). Both sunflower cultivars were the same under water stress and trehalose treatments.

**Figure 2:**
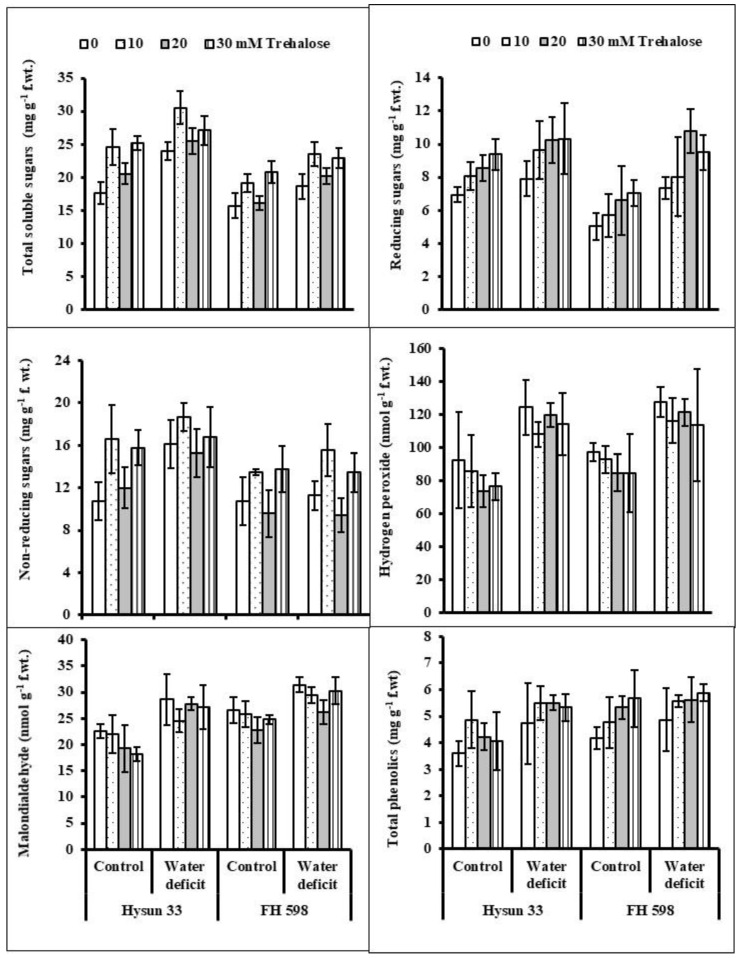
Total soluble sugars, reducing sugars, non-reducing sugars, hydrogen peroxide, malondialdehyde, and total phenolic content of two drought-stressed and non-stressed cultivars of sunflower (*Helianthus annuus* L.) plants subjected to varying levels of foliar-applied trehalose (mean ± S.E.).

The imposition of 60% field capacity markedly increased the levels of reducing sugars and non-reducing sugars as well as the total soluble sugars in both sunflower cultivars. To minimize the negative effects of water stress, the foliage spray of trehalose (10 and 20 mM) was prominent in improving all the above-mentioned attributes of both sunflower cultivars (Table 1; Figure 2). The cv. Hysun 33 was higher in total soluble sugars and reducing sugars under water stress, while both cultivars were uniform in their non-reducing sugars.

Upon exposure to water stress, the MDA and H_2_O_2_ contents increased in both lines of sunflowers (Table 1; Figure 2). A non-significant influence of trehalose supplemented as a foliar treatment was observed under drought stress. The hydrogen peroxide content remained almost the same in both sunflower cultivars, while the MDA concentration was higher in cv. FH 598 than in cv. Hysun 33 under stressed conditions.

An increase was observed in the total phenolics of water-stressed sunflower plants. The foliar spray of trehalose proved non-effective in altering this attribute in both cultivars. In addition, a uniform trend was noticed in all sunflower plants in response to the application of water regimes and trehalose (Figure 2).

**Figure 3:**
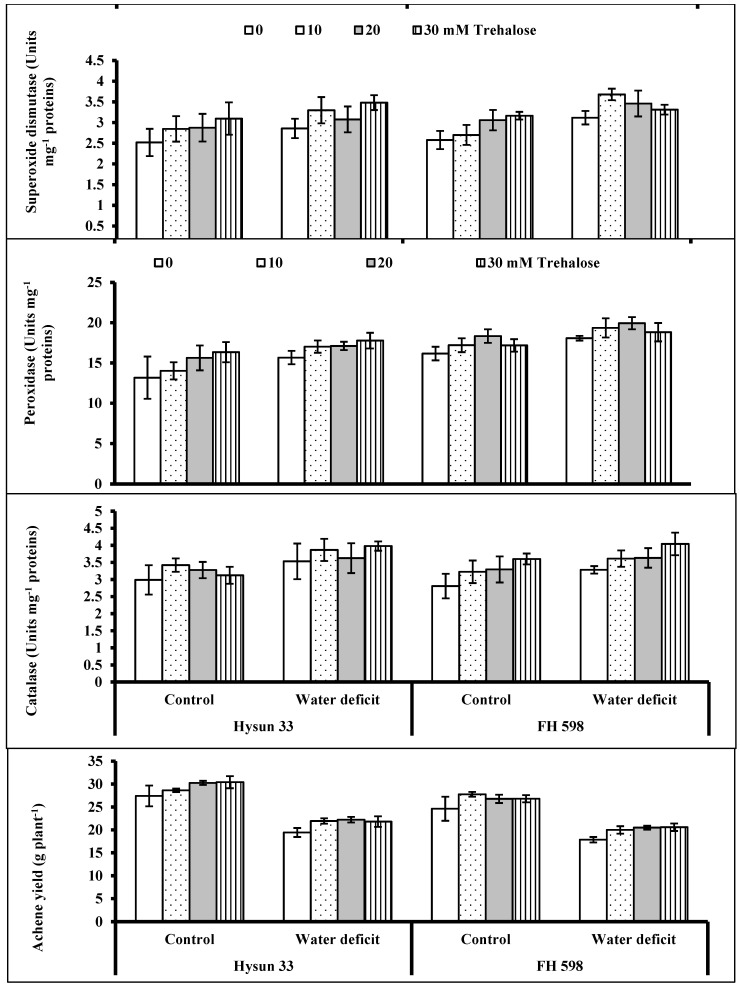
Activities of superoxide dismutase, peroxidase, and catalase enzymes and achene yield per plant for two drought-stressed and non-stressed cultivars of sunflower (*Helianthus annuus* L.) plants subjected to varying levels of foliar-applied trehalose (mean ± S.E.).

The activities of the superoxide dismutase (SOD), peroxidase (POD), and catalase (CAT) enzymes were enhanced in water-stressed sunflower plants (Table 1; Figure 3). The plants treated with trehalose were higher in the activities of POD and SOD enzymes. However, no significant effect of trehalose was found on the activity of catalase. The cv. FH 598 was relatively higher in POD activity, while the cultivars were uniform for the activities of SOD and CAT under water stress.

Water stress induced a significant reduction (20–40%) in the achene yield/plant of all sunflower plants. To combat yield losses, foliar-applied trehalose proved to be significant in improving achene yield by up to 5–10% for both sunflower cultivars under stressed and unstressed conditions. Of both sunflower cultivars, cv. Hysun 33 was better in yield production (Table 2; Figure 3). According to the correlation coefficients (*r*) among different growth, yield, and biochemical attributes, the yield of sunflower plants was significantly correlated with the shoot and root lengths and relative water content, as well as the total soluble proteins (Table S1).

**Figure 4:**
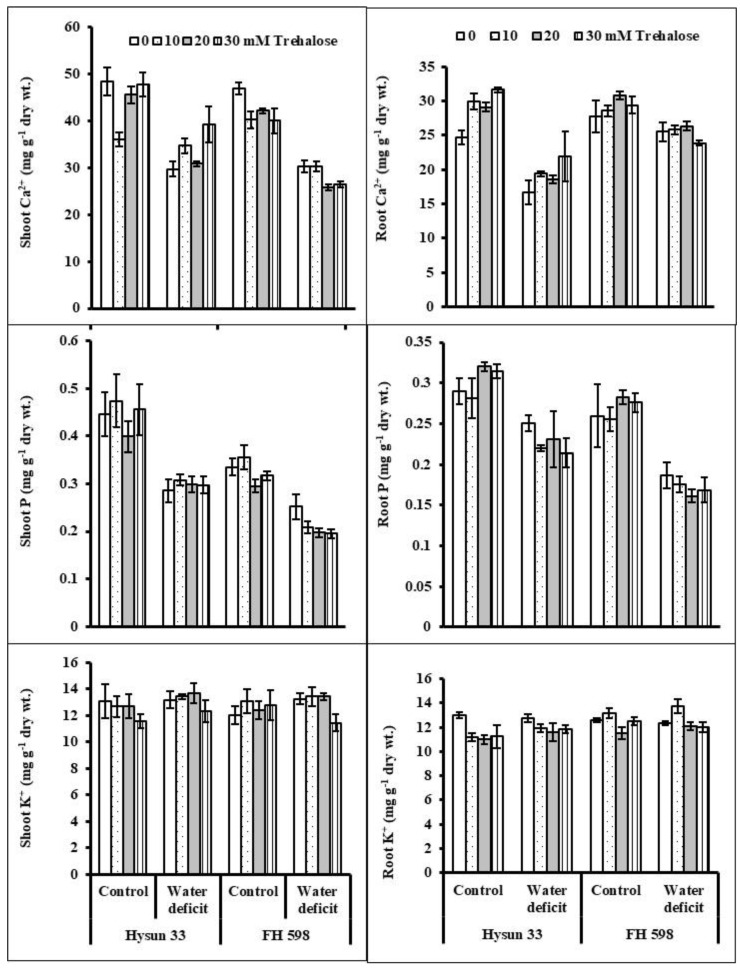
Shoot and root Ca_2_^+^, P, and K^+^ concentrations for two drought-stressed and non-stressed cultivars of sunflower (*Helianthus annuus* L.) plants subjected to varying levels of foliar-applied trehalose (mean ± S.E.).

Water stress significantly decreased the Ca^2+^ and P (shoot and root) concentrations. However, no change was observed in the shoot and root K^+^ concentration under water stress. An exogenous application of trehalose (30 mM) increased the Ca^2+^ concentration (shoot and root), while the accumulation of K^+^ and P (shoot and root) was unaffected. The cv. Hysun 33 was higher in shoot Ca^2+^ and shoot and root P and K^+^ concentrations under water stress (Table 2; Figure 4).

## 3. Discussion

During their whole life span, plants often counteract adverse situations of various environmental stresses, including water desiccation, temperature extremes, quality of irrigation water, humidity, nutritional deficiency, flooding, etc. To combat such harsh environmental conditions, plants tend to change their metabolic and physiological processes to maintain their yield and growth stability [4]. For example, plants tend to accumulate a variety of antioxidants (ascorbic acid, proline, phenolics, glycine betaine, catalase, peroxidase, and superoxide dismutase) as well as various sugars (trehalose, sucrose, glucose, and fructose) in their tissues to cope with stress-induced harmful effects [16]. Many reports show the effectiveness of externally applied sugars to different plants under stress conditions to promote their growth [17,18]. Trehalose, as an important sugar, safeguards biological enzymes and cellular compartments in plants grown under stressful environments [9]. In this study, water stress significantly suppressed the length (shoot and root) of all sunflower plants. However, a foliar spray of trehalose caused the promotion of growth in both cultivars under drought stress. These results are in harmony with what has been reported earlier, wherein trehalose applications promoted the growth parameters of corn [19], radishes [17], and sunflowers [9]. Likewise, the external application of trehalose to *Sinorhizobium saheli* caused growth improvement under PEG-induced osmotic stress [20]. These findings indicated the positive effect of trehalose on plant growth by enhancing their photosynthetic rate, cell division, cell proliferation, antioxidant defense system, and osmoprotective processes of water-deficient plants [9].

The stress-induced damage to biological membranes is the first sign of the adverse effects of stressful environments [21]. In our study, soil conditions that included water scarcity significantly (*p* ≤ 0.001) raised the relative membrane permeability (RMP), but a trehalose application was found to be effective at lowering the RMP of sunflower plants. Likewise, the authors of [22] observed that trehalose-treated water-stressed radish plants showed a reduced RMP in a pot experiment. It has been earlier reported that trehalose could safeguard the membrane structure and integrity of plants upon exposure to stress conditions [23].

The optimal turgor potential of plant tissues is vital for maintaining plant growth processes under stressful cues, and a disruption in the water status [24] of cells is primarily an indication of a stress injury to vital biomolecules and enzymes. In this study, 60% field capacity significantly minimized the RWC of sunflower plants, but exogenously applied trehalose improved the RWC in all water-stressed sunflower plants. The treatments of 10 and 30 mM of trehalose proved to be better at improving this attribute. In some other studies, it has been reported that exogenously applied trehalose enhances the accumulation of key osmolytes which maintaining the turgidity of plant cells [25]. Moreover, the external supplementation of trehalose can also recover intrinsic levels of trehalose to restore the endogenous water potential of plant cells under stress conditions [26].

AsA is believed to accumulate in plant tissues under abiotic stresses and acts as a critical non-enzymatic water-soluble antioxidant [27]. In this study, the concentrations of ascorbic acid were found to be significantly higher at 60% field capacity, but a foliar treatment with trehalose did not alter the levels of AsA in either cultivar. In contrast, a positive effect of externally applied trehalose was observed by Alam et al. [28] in improving the ascorbic acid content in drought-stressed *Brassica* plants. Shafiq et al. [17] also reported the beneficial effect of trehalose applications as a seed pretreatment on the AsA content in water-stressed radish plants. Similarly, trehalose-induced improvement in endogenous AsA was observed in water-stressed maize plants [19] and in the roots of salt-stressed carrot plants [29]. It is highly likely that the elevated levels of ascorbic acid significantly reduced ROS as observed in this experiment.

It is known that during vegetative growth, drought stress may deteriorate several proteins, resulting in reduced levels of cellular proteins [30]. In the same manner, water stress caused the significant suppression of the TSP content in sunflower plants. Moreover, exogenously applied trehalose (20 mM) improved the TSP content of water-stressed sunflower plants. These findings are analogous to those of drought-stressed wheat plants, in which a significant improvement was reported in cellular proteins due to the application of trehalose [31,32]. Radish seed priming with trehalose caused a significant increase in soluble protein levels under drought stress [17]. Under field conditions, Kosar et al. [9] reported a substantial increase in the soluble protein content in trehalose-treated sunflower plants exposed to a limited water supply.

To overcome the effects of stresses, plants tend to accumulate various compatible osmolytes such as sugars, glycine betaine, proline, and other amino acids to adjust their osmotic potential [33]. The stress-induced enhanced accumulation of sugars is an adaptive strategy of plants to maintain the functionality of their metabolic processes [34]. Similar findings were also found in the current study on sunflower plants under water stress. Moreover, trehalose supplementation further raised the levels of total soluble sugars and reducing sugars as well as non-reducing sugars in drought-stressed sunflower plants. Previously, Akram et al. [22] found that priming seeds with trehalose improved sugar levels in water-deficient radish plants. Ibrahim and Abdellatif [32] also reported a marked increase in the soluble sugars of water-stressed wheat seedlings fortified with trehalose. From these results, it can be inferred that higher sugar levels confer water stress tolerance in sunflower plants by maintaining their water relations.

During abiotic stress, the physiological metabolism of plants is significantly perturbed, and in stressed plants, the production of injurious biomolecules such as hydrogen peroxide, malondialdehyde, and other ROS may occur as potential stress indicators [35]. In this trial, the levels of hydrogen peroxide and malondialdehyde were enhanced in the water-stressed sunflower plants. Similar findings have earlier been observed in water-stressed mung bean plants [16]. In this study, the trehalose application did not affect the levels of H_2_O_2_ and MDA in the water-stressed sunflower plants. Analogous to these findings, Kosar et al. [9] exhibited that both the withholding of irrigation water and exogenous trehalose treatments did not affect the ROS generation in sunflower plants under field conditions. Moreover, Akram et al. [22] also found that soaking radish seeds with trehalose did not alter the levels of H_2_O_2_ in radish plants under water-limited conditions. Similarly, Alam et al. [28] found ameliorating effects of trehalose to minimize the H_2_O_2_ content in water-stressed *Brassica* plants. Shafiq et al. [17] reported enhanced MDA levels in water-stressed radish plants, but the application of trehalose minimized the toxic secondary metabolite. Similar studies have been reported for *Brassica* [28] and maize [19]. The decrease in malondialdehyde by trehalose supplementation indicates its defensive role under stressful circumstances to safeguard biological membranes and preserve their dynamic fluidity [36].

Under water stress, the production of ROS is known to cause injurious effects on plant cellular metabolites. To manage such adverse conditions, plants accumulate antioxidants to eliminate the toxic secondary metabolites and maintain their growth [37]. The activities of enzymatic antioxidants (SOD, CAT, and POD) were found to be significantly enhanced under moisture-deficit stress. This is analogous to water-stressed corn [38], wheat [39], and sunflowers [9]. Exogenously supplemented trehalose induced a considerable increase in the activities of enzymatic antioxidants in the sunflower plants. These results are in agreement with those of Rohman et al. [37], wherein a trehalose treatment was reported to induce an improvement in the activities of CAT and SOD, but suppress that of POD, in salt-stressed maize pants. Catalase is reported to detoxify H_2_O_2_ more than any other antioxidant enzyme during oxidative stress [40]. Such findings have already been observed under various environmental stresses in *Brassica* plants [28], radishes [22], wheat [32], and sunflowers [9]. These results indicate that trehalose plays a central role in suppressing the levels of toxic secondary metabolites in plant cells under oxidative stress.

Some of the key minerals in plant tissues are known to improve plant resistance against pervasive abiotic stresses. In the case of desiccated soil conditions, plants fail to uptake an optimal quantity of nutrients, which causes adverse effects on the vegetative growth and overall crop yield [41]. In the present investigation, moisture-deficit conditions caused a marked decrease in the accumulation of shoot and root Ca^2+^ and P, but shoot and root K^+^ concentrations remained unaffected. Foliar-applied trehalose proved to be useful for improving the uptake of shoot and root Ca^2+^ and K^+^ in sunflower plants under water-deficit conditions. Similarly, Akram et al. [22] indicated improvement in shoot P in water-stressed radish plants raised from seeds treated with trehalose. In another study, rice plants pre-treated with trehalose showed a reduced Na^+^/K^+^ ratio [42].

In our study, water-deficit conditions caused a significant reduction in the achene yield per plant for both cultivars of sunflower plants. Similar findings have been earlier reported for other crops grown under drought stress conditions, e.g., ajwain [43], wheat [44], and sunflowers [9]. There could be several reasons for a drought-stress-induced decline in growth and yield, such as damage to the photosynthetic apparatus, the increased generation of ROS, or nutritional and hormonal imbalances, as well as many others. The trehalose fortification improved the achene yield per plant for the sunflower plants in the current study, and it was significantly correlated with the shoot and root length, RWC, and the total soluble proteins. Therefore, these attributes can be suggested to be involved in the drought stress tolerance of sunflower plants.

## 4. Materials and Methods

An experiment was arranged during the spring season (February to April 2015) to determine the influence of foliar-applied trehalose on the pre-flowering stage and yield parameters of sunflowers (*Helianthus annuus* L.). Two cultivars of sunflowers (FH 598 and Hysun 33) were used for the experimentation. Each plastic pot (diameter of 21 cm and 24.5 cm in height) was filled with sandy loam soil (7.0 kg). Distilled-water-soaked sunflower achenes were planted in each pot with the hand-drill method. The experimental design was a three-factor factorial: factor 1, drought (2 levels); factor 2, trehalose (4 levels); and factor 3, cultivars (2 levels). There were 3 (pots) replicates for each treatment. Five plants were maintained in each pot by thinning after one week of germination. After 21 days of vegetative growth, drought stress (60% field capacity) was initiated. Irrigation was performed as per the drought requirements, considering the respective field capacities. After one month of drought stress, varying (0, 10, 20, and 30 mM) levels of trehalose were foliar-applied at the pre-flowering stage. After two weeks of trehalose sprays, the data were collected for the attributes described below. For the yield attributes, two plants were kept growing until flowering maturation.

### 4.1. Shoot and Root Length

The length of the shoots and roots of the plants were determined using a measuring scale.

### 4.2. Relative Membrane Permeability (RMP)

For recording initial EC_o_, the youngest fully expanded leaves were dipped in each sample in a test tube with 20 mL dH_2_O, after which time all tubes were vortexed for 5 s. For measuring EC_1_, the samples were placed overnight at 4 °C, whereas for recording the final electrical conductivity (EC_2_), the test tubes were autoclaved as described in the assay performed by Yang et al. [45].

### 4.3. Relative Water Content (%)

The fresh weight of each leaf sample was measured, and then each leaf was immersed in distilled water for 3 h before determining the turgid weight. The samples were then placed in an oven for five days to measure the dry weight, as described by Barrs and Weatherley [46].

### 4.4. Ascorbic Acid (AsA)

An amount of 0.5 g of leaf material from each sample was ground in 10 mL of trichloroacetic acid (6% *w*/*v*). After filtration, 2% diphenyl-hydrazine, thiourea, and 80% H_2_SO_4_ were added to the filtrate as described by Mukherjee and Choudhuri [47]. Finally, the optical density (OD) of each treated sample was noted at 530 nm to calculate the AsA content.

### 4.5. Sugar Content

Fresh leaf tissue (each 0.1 g) was homogenized in ethanol (80%, 10 mL). Then, mixing, heating, and cooling were applied according to the protocol of Yemm and Willis [48] for the estimation of total soluble sugars. Similarly, the filtrate was used for the estimation of reducing sugars by adopting the procedure of Nelson [49]. However, the procedure of Loomis and Shull [50] was followed for the appraisal of non-reducing sugars.

### 4.6. Hydrogen Peroxide (H_2_O_2_)

About 0.5 g of fresh leaf tissue was ground in 5 mL of trichloroacetic acid (TCA, 0.1%). The extract was centrifuged, and further steps for processing the sample’s supernatant were completed according to the procedure of Velikova et al. [51].

### 4.7. Malondialdehyde (MDA)

The trituration of the leaf (each 0.5 g) was carried out using 3 mL of TCA. The MDA content of the aliquot was determined by following the procedure described by Cakmak and Horst [52]. The OD of all samples was noted at 532 and 600 nm.

### 4.8. Total Phenolics

A leaf (0.5 g) was homogenized in an ice bath using 5% acetone. The protocol of Julkenen-Titto [53] was employed to estimate the total phenolic content.

### 4.9. Total Soluble Proteins

A leaf sample (each 0.5 g) was extracted with ice-cold phosphate buffer. Then, 2 mL of the Bradford reagent was mixed with 1 mL of the sample mixture in a test tube. The total soluble protein content was determined by following the methodology of Bradford [54].

### 4.10. Estimation of Antioxidant Enzymes

A leaf (0.5 g) was extracted in 10 mL of sodium phosphate buffer (pH 7.8). The activity of superoxide dismutase (SOD) was determined by following the methods of van Rossum et al. [55]. The activity of the peroxidase (POD) enzyme was determined by adopting the procedure described by Chance and Maehly [56]. Moreover, the activity of the catalase (CAT) enzyme was determined by following the methods of Luck [57].

### 4.11. Inorganic Nutrients

Dried samples of sunflower roots and stems (0.15 g each) were put in glass digestion flasks, each containing 2 mL H_2_SO_4_. All tubes were kept at room temperature overnight. Then, these samples were transferred to a hot plate set at 350 °C. After 10–15 min of heating, the samples turned yellow. By adding 1–2 drops of H_2_SO_4_ to each reaction mixture, they became colorless, reflecting the endpoint of digestion. The final volume was adjusted to 50 mL with dH_2_O for the determination of the following inorganic elements:

### 4.12. Determination of Potassium (K^+^) and Calcium (Ca^2+^)

The concentrations of K^+^ and Ca^2+^ were determined using a flame photometer by utilizing A-grade standards. The concentrations of these nutrients were determined from their respective standard curves.

### 4.13. Determination of Phosphorus (P)

For the phosphorus determination, the Barton reagent was prepared from solution A and solution B as instructed by Jackson [58]. In a test tube, 1 mL of the diluted digestion mixture was treated with 2 mL of Barton reagent, and finally, the volume was adjusted to 25 mL using distilled water. After one hour, the samples were read at 470 nm. The phosphorus concentration in all samples was calculated using a standard curve.

### 4.14. Achene Yield

Upon the ripening of the sunflower heads, the achenes from each plant were segregated. The achenes were air-dried and the weight per plant was recorded using a top-loading balance.

### 4.15. Statistical Analysis

The ANOVA test was employed to test the probability of all treatments. In addition, means were compared at the 5% LSD level.

## 5. Conclusions

Foliar-supplemented trehalose significantly increased the plant growth, RWC, proteins, sugars, activities of enzymatic antioxidants, shoot and root Ca^2+^, root K^+^, and achene yield of plants for both sunflower cultivars. Cv. Hysun 33 had better ascorbic acid levels, total soluble sugars, non-reducing sugars, and shoot and root P, whereas cv. FH 598 was relatively better in its RMP, MDA content, peroxidase activity, and root Ca^2+^. As a whole, the exogenous application of trehalose improved the physio-biochemical characteristics and achene yield of drought-stressed sunflower plants. Therefore, the foliar application of trehalose can be suggested for improving the drought stress tolerance potential in different plants.

## Figures and Tables

**Table 1:** Mean square data for different physio-biochemical attributes of sunflower (*Helianthus annuus*) plants subjected to varying (0, 10, 20, and 30 mM) levels of trehalose under drought stress.

**Source of Variation**	**df**	**Shoot Length**	**Root Length**	**RMP**	**RWC**	**Ascorbic Acid**	**Total Soluble Proteins**
Drought (D)	1	3291 ***	135.1 ***	2105 ***	611.6 ***	15.35 ***	3205 ***
Trehalose (Tre)	3	190.7 ***	4.348 *	244.1 ns	89.68 **	1.821 ns	1570 ***
Cultivars (Cvs)	1	83.26 *	40.64 ***	981.9 **	78.48 *	46.47 ***	53.93 ns
D × Tre	3	14.18 ns	1.015 ns	240.9 ns	15.03 ns	0.367 ns	1139 ns
D × Cvs	1	9.765 ns	17.01 ***	20.55 ns	85.27 *	0.389 ns	1334 ns
Tre × Cvs	3	8.807 ns	0.515 ns	48.35 ns	7.092 ns	1.276 ns	281.2 ns
D × Tre × Cvs	3	16.30 ns	2.390 ns	99.87 ns	10.98 ns	0.648 ns	363.3 ns
		**Total Soluble Sugars**	**Reducing Sugars**	**Non-Reducing Sugars**	**Hydrogen Peroxide**	**MDA**	**Total Phenolics**
Drought (D)	1	266.5 ***	67.85 ***	3794 ***	1649 ***	470.5 ***	9.824 *
Trehalose (Tre)	3	111.9 ***	18.80 *	489.3 *	531.3 ns	29.53 ns	2.872 ns
Cultivars (Cvs)	1	359.1 ***	30.08 *	250.2 ns	466.8 ns	185.0**	4.087 ns
D × Tre	3	7.674 ns	1.466 ns	247.7 ns	232.0 ns	12.26 ns	0.0288 ns
D × Cvs	1	8.265 ns	9.292 ns	822.5 *	81.78 ns	18.55 ns	1.329 ns
Tre × Cvs	3	4.730 ns	1.166 ns	119.4 ns	14.07 ns	11.85 ns	0.8396 ns
D × Tre × Cvs	3	2.401 ns	0.537 ns	188.6 ns	26.96 ns	7.537 ns	0.315 ns
		**Superoxide Dismutase**	**Peroxidase**	**Catalase**			
Drought (D)	1	2.964 ***	61.67 ***	3.672 ***			
Trehalose (Tre)	3	0.719 *	12.68 *	0.803 ns			
Cultivars (Cvs)	1	0.261 ns	84.04 ***	0.025 ns			
D × Tre	3	0.167 ns	1.058 ns	0.069 ns			
D × Cvs	1	0.119 ns	0.316 ns	0.075 ns			
Tre × Cvs	3	0.075 ns	3.232 ns	0.218 ns			
D × Tre × Cvs	3	0.099	0.272 ns	0.035 ns			

ns = non-significant; *, **, and *** = significant at a level of 0.05, 0.01, and 0.001, respectively.

**Table 2:** Mean square data for mineral nutrients and achene yield attributes of sunflower (*Helianthus annuus*) plants subjected to varying (0, 10, 20, and 30 mM) levels of trehalose under drought stress.

**Source of Variation**	**df**	**Shoot Ca^2+^**	**Root Ca^2+^**	**Shoot K^+^**	**Root K^+^**
Drought (D)	1	2495 ***	244 ***	3.672 ns	1.084 ns
Trehalose (Tre)	3	4533 *	2940 **	4.198 ns	4.394 ***
Cultivars (Cvs)	1	2245 ***	1700 ***	0.173 ns	7.333 **
D × Tre	3	1117 ***	526.7 ns	1.205 ns	0.770 ns
D × Cvs	1	4435 *	14,241 ***	0.444 ns	0.390 ns
Tre × Cvs	3	8907 ***	2876 **	0.437 ns	3.686 **
D × Tre × Cvs	3	2038 ns	218.1 ns	1.809 ns	0.270 ns
		**Shoot P**	**Root P**	**Achene Yield/Plant**	
Drought (D)	1	0.2674 ***	0.112 ***	851.3 ***	
Trehalose (Tre)	3	0.004 ns	0.0007 ns	24.98 ***	
Cultivars (Cvs)	1	0.163 ***	0.031 ***	74.54 ***	
D × Tre	3	0.002 ns	0.002 ns	0.057 ns	
D × Cvs	1	0.004 ns	0.002 ns	4.574 ns	
Tre × Cvs	3	0.001 ns	0.0002 ns	1.095 ns	
D × Tre × Cvs	3	0.001 ns	0.0001 ns	2.256 ns	

ns = non-significant; *, **, and *** = significant at a level of 0.05, 0.01, and 0.001, respectively.

## Supplementary Materials

The following supporting information can be downloaded at: https://www.mdpi.com/article/10.3390/plants11202780/s1, Table S1: Correlations coefficients (r) among different growth, yield and biochemical attributes of drought-stressed sunflower (*Helianthus annuus* L.) plants treated with trehalose as a foliar spray.
