# Supplementary material for: Trehalose-Induced Regulations in Nutrient Status and Secondary Metabolites of Drought-Stressed Sunflower (Helianthus annuus L.) Plants
Source: Plants (Basel). 2022 Oct 20;11(20):2780. doi: 10.3390/plants11202780 (PMC9607548; doi:10.3390/plants11202780)
Supplement: Supplementary file 1 [file plants-11-02780-s001.zip › plants-1922029-supplementary.pdf]

Table S1: Correlations coefficients (*r*) among different growth, yield and biochemical attributes of drought-stressed sunflower (*Helianthus annuus* L.) plants treated with trehalose as a foliar spray.

Parameters	Shoot length	Root length	RMP	RWC	AsA	TSP	TSS	RS	NRS	H_2O_2	MDA	T. Phenolics	SOD	POD	CAT	Achene yield
Shoot length		0.62*	-0.384	0.621*	-0.320	0.524*	-0.267	-0.253	-0.122	-0.45	-0.371	-0.071	-0.316	-0.258	-0.263	0.722**
Root length			-0.572	0.284	-0.022	0.485	-0.050	-0.113	0.044	-0.446	-0.450	-0.271	-0.290	-0.390	-0.290	0.744**
RMP				-0.147	-0.271	-0.280	-0.064	0.126	-0.130	0.141	0.261	0.057	0.085	0.289	-0.006	-0.426
RWC					-0.300	0.419	-0.145	-0.172	-0.028	-0.353	-0.254	-0.110	-0.233	-0.02	-0.113	0.523*
AsA						-0.155	0.402	0.358	0.171	0.010	-0.126	0.075	0.269	-0.060	0.302	-0.142
TSP							-0.085	-0.148	0.014	-0.358	-0.274	0.012	-0.315	-0.013	-0.122	0.485*
TSS								0.409	0.81**	0.223	0.079	0.145	0.208	-0.041	0.360	-0.164
RS									-0.175	0.069	-0.051	0.241	0.292	0.204	0.206	-0.208
NRS										0.208	0.114	-0.02	0.052	-0.177	0.250	-0.040
H_2O_2											0.379	0.123	0.316	0.209	0.188	-0.516
MDA												0.378	0.117	0.223	0.189	-0.552
T. Phenolics													0.271	0.304	0.371	-0.189
SOD														0.434	0.384	-0.262
POD															0.360	-0.331
CAT																-0.293
Achene yield																

Abbreviations: RMP, relative membrane permeability; RWC, relative water content; MDA, malondialdehyde; H_2O_2, hydrogen peroxide; T. Phenolics, total phenolics; AsA, ascorbic acid; CAT, catalase; POD, peroxidase; SOD, superoxide dismutase; TSS, total soluble sugars; RS, reducing sugars; NRS, non-reducing sugars; TSP, total soluble proteins; Achene yield/plant; *, **, significant at 0.05 and 0.01 levels; ns, no significant.
